# Taxation without Representation

**Published:** 1888-03

**Authors:** 


### EDITORIAL DEPARTMENT.

F. E. DANIEL, M. D., EDITOR AND PUBLISHER

THIS JOURNAL, by unanimous vote of the members, was made the Official Organ of the Austin District Medical Society.

COLLABORATORS:

*Dr. R. M. Swearingen, Austin.*

*Dr. B. E. Hadra, Austin.*

*Dr. Geo. Cupples, San Antonio.*

*Dr. T. C. Osborn, Cleburne, Texas.*

*Dr. E. J. Doering, Chicago.*

*Dr. Odo Betz, Germany.*

*Dr. J. W. McLaughlin, Austin.*

*Dr. T. J. Tyner, Austin.*

*Dr. J. F. Y. Paine, Galveston.*

*Dr. E. Goldmann, San Jose, Cal.*

*Dr. H. O. Marcy, Boston.*

*Dr. E. Meierhof, New York.*

*E. J. Beall, M. D., Fort Worth, Texas.*

### "TAXATION WITHOUT REPRESENTATION."

### DR. TALLEY'S LETTER.

We are glad to see that our article on local societies paying tribute to the State Medical Association, is attracting attention. The tax should be abolished. It will be seen that Dr. Tally has quite misunderstood us with regard to the majority not being represented. Of course, an officer is elected by a majority *of those who vote*—or are permitted to vote. We mean that the largest part of the profession in affiliation with the State association is not represented, by delegate, *in the nominatiug committee*, and it seems to us quite clear that they are not. Take, as we did, Travis county Medical Society for an illustration. This county society has forty members, and sends eight delegates and pays $20 annually; Blank county, for instance, has a medical society with ten members and sends two delegates, paying five dollars tax. Each society is represented in the nominating committee by *one delegate* who casts *one vote* in the choice of officers. It is not clear that the majority of the members of Travis county are not represented—have no voice? Or, take a remote county, sparsely settled, with *no* organization and only two or three resident physicians. This county is represented in the nominating committee by *one* and the casts *one* vote and pays *no* tax—the one delegate or representative not being a member of the association. We hold also that each delegate should vote on all questions before the house; if absent, call roll by counties and cast the vote by county or society representation. We think the plan of having local societies—whose members are mostly, perhaps, members of the State association—pay 50 cents *per capita* of its members, unjust, and should be abolished.

Again, Dr. Talley takes umbrage at the word "iniquitous." We did say, this "iniquitous clause," and we hold that anything is "iniquitous" that leads to, or causes, or results in evil, or—injustice. But the Doctor quite overlooks the fact that our fulmination *was* directed to "*the workings*" of the association "*no matter what the letter of the law may be*"—and *not* to the constitution and by-laws—and the "election of officers," was especially referred to.

We are glad to see that although the Doctor "pitches into us" in the beginning of his letter, he comes around all right and agrees with us in the end. We, too, want to see a change in our elections, and if we are not mistaken, the new constitution and by-laws which will be submitted in April, provide for election in the meeting without nomination. This double voting—one man's having one, another having two votes, necessarily leads to confusion, and we, for one, shall advocate its abolition, for it is not carried out, and we do not believe that Dr. Talley can cite a single instance where any one ever did "cast two votes" in any meeting of the association.

